# Safe menstrual hygiene management practice and associated factors among female adolescent students at high schools in central Ethiopia: A mixed–method study

**DOI:** 10.3389/fpubh.2022.913262

**Published:** 2022-07-26

**Authors:** Berhanu Senbeta Deriba, Girma Garedew, Diriba Gemeda, Tinsae Abeya Geleta, Kemal Jemal, Elias Teferi Bala, Mulugeta Mekuria, Tadesse Nigussie, Dejene Edosa Dirirsa, Elsabeth Legesse

**Affiliations:** ^1^Department of Public Health Fitche, College of Medicine and Health Sciences, Salale University, Fiche, Ethiopia; ^2^Department of Nursing, College of Medicine and Health Sciences, Salale University, Fitche, Ethiopia; ^3^Department of Public Health, Ambo University College of Medicine and Health Sciences, Ambo, Ethiopia; ^4^Department of Midwifery, College of Medicine and Health Sciences, Salale University, Fitche, Ethiopia

**Keywords:** adolescent girls, central Ethiopia, hygiene, menstruation, school

## Abstract

**Background:**

Menstrual Hygiene Management (MHM) is a much-neglected issue in developing countries, including Ethiopia. Menstruating women and girls are forced into isolation, prevented from movement, dietary restrictions, and can be prevented from participating in daily routine activities. Furthermore, the way almost all previous studies conducted in Ethiopia measured the practice of MHM did not meet standard definition of safe MHM. This study aimed to assess safe management of menstrual hygiene practice and associated factors among female adolescent students in public high schools in central Ethiopia.

**Methods:**

A mixed-methods approach was employed in this study. Systematic random sampling technique was used to select 846 study participants. The collected data were entered through EPI INFO version 7 and exported to SPSS version 23 for cleaning and analysis. Bivariate and multivariate logistic regression analysis were performed to identify the association between MHM and independent variables. Finally, AOR, 95% CI, and *p*-value < 0.05 were considered statistically significant. The qualitative data was analyzed by ATLAS.ti in order to extract the main themes and categories. Direct quotations were presented with a thick description of the findings.

**Results:**

The safe management of menstrual hygiene was 28.20%. Living with parents (AOR = 2.51, 95% CI:1.11–5.68), living with relatives (AOR = 7.41, 95% CI:2.55–21.54), having a merchant mother (AOR = 1.81, 95% CI:1.14–2.9), having a mother who has private work (AOR = 4.56, 95% CI:1.31–5.90), having a farmer father (AOR = 1.53, 95% CI:1.1–2.31), rural resident (AOR = 1.61, 95% CI: 1.17–2.21) and realizing the absence of container for storing sanitary napkins in the toilet of the school latrine (AOR = 1.44, 95% CI: 1.1–0.94) were factors associated with MHM. Findings from a qualitative study were discussed under four themes to explore barriers to menstrual hygiene management, and three themes emerged as enablers to menstrual hygiene management.

**Conclusions:**

The safe management of menstrual hygiene was low among adolescent girls. People with whom adolescent girls live, the occupational status of mother and father, residence, the availability of a container to dispose of sanitary napkins in school toilets were factors associated with menstrual hygiene management. Behavioral change communications must be provided to female students about menstrual hygiene.

## Introduction

Menstruation is a normal natural process through which millions of women and girls across the world pass-through each month (1). Menstrual hygiene management is a process in which women and adolescent girls use clean menstrual management material to absorb or collect blood that can be changed in privacy room for the duration of menstrual time. Menstrual management includes use of soap and water to wash the body as necessary, and have access to facilities to dispose of the menstrual management materials (2, 3). Menstrual hygiene management (MHM) is a very neglected issue in developing countries, including Ethiopia due to the lack of female involvement in decision making, lack of social support, lack of access to products and facilities, lack of information, and awareness (4, 5).

In society, menstruation is viewed as something contaminated or dirty (6). Adolescent girls, especially those in rural areas, face poor menstrual hygiene management practice in developing countries (7–10). Although menstruation is a normal process, it is regarded as too taboo and many negative cultural attitudes associated with it, such as the idea that menstruating women and girls are contaminated, dirty, and impure. Research reported that menstruating girls face shame, fear, confusion, and poor MHM due to inadequate information, absence of social support, ongoing social and hygiene taboos, and lack of water, sanitation, and waste disposal facilities in school environment (8, 9, 11–13). Schools in developing countries lack adequate facilities for water, sanitation, and hygiene (WASH) facilities which contributes to poor menstrual hygiene practices for girls (9, 10, 14, 15).

Access to MHM is fundamental to achieve the Sustainable Development Goals (SDGs) (8). Lack of basic knowledge and MHM can lead to urinary tract infection, stress, early, and unwanted pregnancy, all affecting girl's health. Girls may be absent, less attentive at school during menstruation due to a lack of WASH facilities or lack of support from the school community, affecting educational performance or work performance, affecting economic opportunities (10). When taboos and myths prevent menstruating women and girls from full participation in society, gender equality cannot be guaranteed. Furthermore, inability to develop markets for quality menstrual materials can affect sustainable consumption and production patterns (16). Lack of access to clean and effective absorbents, lack of access to soap and water, lack of privacy, inadequate facilities to change, clean and dispose of absorbents are some of the challenges associated with effective MHM (8–11, 17). Menstrual hygiene practice is influenced by different factors, including age, educational status of adolescents, educational status of adolescent's father and mother, family size, residence, living arrangements, family monthly income, lack of WASH facilities, lack of latrine privacy, knowledge of women about menstruation and fearing of teasing by boys (7, 18–30). Different studies were conducted on the topic of knowledge and practice of menstrual hygiene in Ethiopia through collecting data from only menstruating women. However, good MHM needs the collaboration of at least teachers and girls themselves. To obtain rich information about MHM, it is important to include teachers, girls, and observe WASH facilities during data collection. Therefore, this study aimed to safe assess menstrual hygiene management practice and associated factors among female adolescent students in public high schools in central Ethiopia.

## Methods

### Study design, area, and period

A mixed-methods approach, consisting of an institution-based cross-sectional study and a qualitative study, was conducted among female adolescent students at six randomly selected public high schools in the North Shewa zone, central Ethiopia. North Shewa Zone is located at 112 kilometers to the north of Addis Ababa. North Shewa Zone has a total population of 1.6 million. In the Zone there is one public university, 705 primary schools, and 40 public highs schools. The study was conducted from January 1 to February 30, 2021.

### Population

All female adolescent students who were attending public high schools in the North Shewa during the data collection period were the source population. Students who were randomly selected and included in the study were the study population. The qualitative study participants were selected purposively. Accordingly, the in-depth interview and key informant interviews included adolescent girls aged >14 years old, school directors, school teachers, and a school girl club representative to get in-depth information about the practice of menstrual hygiene management.

#### Inclusion and exclusion criteria

All 14–19-years-old female adolescents who started menses and who were willing to participate in the study were included in the study. Female adolescents who had no history of menstruation before data collection day were excluded from the study.

### Sample size determination

For the quantitative study sample size was calculated using a single population proportion formula using proportion of safe management of menstrual hygiene management practice of 46.4% from the study conducted in Ambo town (22). Taking into account the 95% confidence level and 5% marginal error, after adding the 10% non-response rate, the total sample size was 422. After multiplying 422 by the design effect or 2, the final sample size was 844. For the qualitative study, observation of WASH facilities, in-depth interviews (IDI), and key informant interviews (KII) were conducted to address the qualitative study part. Accordingly, 12 IDI and 12 KII were conducted for this purpose.

### Sampling procedure

The study was carried out in six randomly selected high schools in the north Shewa zone. Multistage sampling was used to select schools and classes. The number of study participants was proportionally allocated to each high school based on the number of adolescents who were attending at each high school, which was obtained from each high school registrar office (Fitche-197, Garba guracha-150, Debra tsige-126, Muka turi-143, Fital-116 and General Tedase Biru-114 students). Finally, the study participants were selected using a systematic random sampling technique using student registration as the sampling frame. For the qualitative study, in-depth interviews and key informant interviews were conducted with participants who were selected through a judgmental sampling method. The observation of WASH facilities in each school, 12 in-depth interviews with female students, six key informants interviews with school administrators (one at each school), and six key informant interviews with the focal person of the girls' clubs (one at each school) were conducted.

### Data collection tool and procedure

Data were collected through a self-administered interview using a standardized questionnaire developed from previous similar studies and UNICEF (18, 26, 31). A structured and pre-tested questionnaire was used for data collection. The questionnaire consists of questions that assessed the sociodemographic characteristics, knowledge, practice of menstrual management, school absenteeism status of students, and school WASH facilities. The questionnaire was prepared in English and translated into the Afan Oromo language for better understanding for both data collectors and respondents, and translated back to the English version to verify consistency.

Six BSC nurses were recruited for data collection. Three midwives were also recruited for supervision. Qualitative data were collected through key informant interviews, in-depth interviews and observation that were conducted with students, school directors, and the focal person of girls' clubs to assess the menstrual hygiene related issues of adolescents, teachers and school settings. Face-to-face interviews were held using a semi-structured interview guide, prepared in English, and translated into Afan Oromo and Amharic. In-depth interviews and key informant interviews were conducted by the principal investigator through note-taking and tape-recording. Interviews were conducted at a covenant time selected by participants. An observational check list was used to identify the status of WASH facilities in schools including the availability of separate latrines and the number of latrines for men and women, the availability of water around the latrine, the cleanliness of the toilet and privacy, the availability of waste disposal bins around the latrine, the availability of water, soap and toilet paper was evaluated. The observational check list was taken from UNICEF and modified accordingly (5).

### Data quality assurance

Three days of training was given to data collectors and supervisors on the objective of the study, the contents of the questionnaire, confidentiality, the right of respondents, and how to collect data. pretest was conducted on 5% of the sample at Dagam High School which was not included in the main study. To maintain the validity of the data collection instruments, data collectors and supervisors discussed on the questionnaire and modified for inconsistencies and ambiguity before the actual data collection. Close supervision was conducted during data collection. Data were entered into the computer twice and consistency was checked during data entry. For the qualitative study: the data collection methods were triangulated. Specifically, the qualitative data collection method and quantitative data collection methods were used together to get in-depth information about menstrual hygiene-related issues. During the data collection process, the researcher used recorded materials (tape-recorder) and written field notes. The qualitative data coding and analysis were checked by qualitative experts. A member check was done by the participants to check and verify the interpretations and findings from the qualitative study.

### Operational definitions

#### Unsafe menstrual hygiene practice

Menstrual hygiene practice is considered **unsafe** when it does not meet any of the following four criteria, but it is **safe** if it meets all of them. These four criteria include (1) if the woman uses safe absorbents (absorbents are considered safe if they are commercially available sanitary pads called modes or new clothes that are locally available). Old clothes and other items such as toilet paper, mattress, sponge, or underwear alone are considered unsafe, (2) changing absorbents two or more times per 24 h, (3) if the girl wash her genitalia two or more times per day, and (4) if the girls dispose of the used menstruation pad by burying or burn it after use (27, 32).

### Data processing and analysis

For quantitative study, the data were checked for completeness, cleaned and entered in EPI INFO version 7 and exported to SPSS version 23 for data cleaning and analysis. Tables and pie charts were used to present results. The goodness-of-fit model (Hosmer and Lemshow) was used for the fitness of the model. Bivariate and multivariate logistic regression analysis was performed to see the association between MHM and independent variables. Variables with a *p*-value < 0.25 at bivariate logistic regression were entered into multivariate logistic regression. Finally, AOR with 95% CI and *p*-value < 0.05 were used to declare a statistically significant association. The qualitative study data were first transcribed verbatim. The next step was to translate the transcript from the local languages (Afan Oromo and Amharic) into the English language. The transcript was copied to ATLAS.ti version 7 for analysis. Then ATLAS.ti version 7 was used for developing categories and themes. The researchers conducted qualitative data analysis using inductive thematic analysis, which aimed to identify a set of main themes that captured the diverse views and feelings expressed by participants. Direct quotations were presented with a thick description of the findings to triangulate the quantitative results.

### Ethical considerations

The study was carried out after obtaining ethical approval from the Ethics Review Committee of Salale University. The support letter was obtained from the education office of the North Shewa Zone. School directors were informed of the objective of the study and permission was obtained to conduct the study from six participating schools. Written consent was obtained after confirming the objective and purpose of the study for all study participants who were over 18 years of age and from parents or guardian for those participants who were under 18 years of age. The privacy and confidentiality of the study participants were also strictly maintained.

## Results

### Sociodemographic characteristics

A total of 840 students participated in this study, making a response rate of 99.52%. The mean age of the study participants was 17.64 ± 1.24 years and almost all, 93.1% of the respondents were in the age groups of 16–19. About two-thirds of the students (69.9%) were from rural areas. Five hundred and seventy-five (68.5%) of the students live with their both parents. Two hundred and sixty-two women of the students had a farmer father. More than half (55%) of the female students did not obtain regular pocket money from their parents (Table 1).

**Table 1:** Socio demographic characteristics of female adolescents in public high schools in the North Shewa zone, Oromia regional state, central Ethiopia, 2021.

**Variable**	**Frequency**	**Percentage (%)**
**Residence**		
Urban	253	30.1
Rural	587	69.9
**Age groups**		
13–16	58	6.9
16–19	782	93.1
**Grade of students**		
Grade 9	82	9.8
Grade 10	119	14.2
Grade 11	216	25.7
Grade 12	423	50.4
**Educational level of father**		
Have no formal education	474	56.4
Have formal education	366	43.6
**Educational level of mother**		
Have no formal education	349	41.5
Have formal education	491	58.5
**Occupation of mother**		
Housewife	366	43.6
Student	8	1
Merchant	152	18.1
Private work	28	3.3
Employee	70	8.3
Daily laborer	60	10.7
Farmer	126	15
**Occupation of father**		
Merchant	188	22.4
Private work	36	4.3
Employee	148	17.6
Driver	36	4.3
Daily laborer	170	20.2
Farmer	262	31.2
**With whom do you live?**		
Both parents	575	68.5
Mother only	148	17.6
Relatives	74	8.8
Father only	16	1.9
Living alone	27	3.2
**Obtain regular pocket money from their parents**		
Yes	378	45
No	462	55

### Practice of menstrual hygiene management

**Figure 1:**
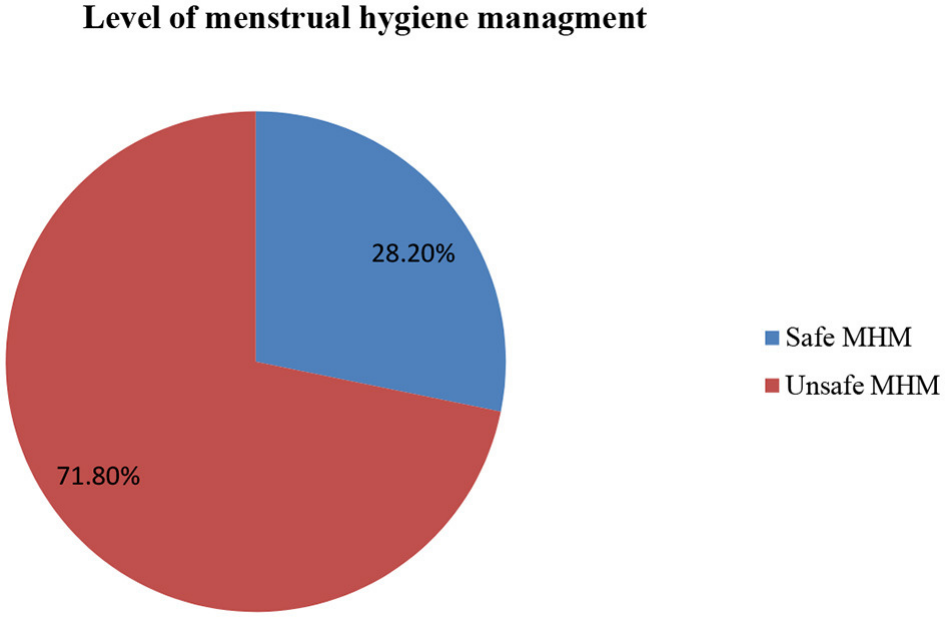
Level of MHM practice among adolescent students in the North Shewa Zone, Ethiopia, 2021.

Most (89.6%) of the respondents used sanitary pads to absorb menstrual blood. Half, 50.2% of the study participants have changed absorbent materials more than twice per day. Three-fourths (74.2%) of the female students washed their genitalia more than two times a day. Five hundred and eighteen (61.7%) washed their genitalia with water and soap. Three hundred twenty-seven (38.9%) of the respondents disposed of the absorbent materials used by putting them in waste bins/burying or burning (Table 2). Regarding the general management of menstrual hygiene, 603 (71.8%) of the students had unsafe MHM practice, while only 237(28.2%) had safe MHM practice (Figure 1).

**Table 2:** Management of menstrual hygiene among female adolescents in public high schools in the north Shewa Zone, Oromia regional state, central Ethiopia, 2021.

**Variable**	**Frequency**	**Percentage (%)**
**Materials used to absorb menses**		
Disposable sanitary pads	753	89.6
New cloth pieces	50	6
Old cloth pieces	26	3.1
Others^a^	13	1.3
**Frequency of changing absorbent per day**		
Once	121	14.4
Twice	422	50.2
Three times	239	28.6
More than four times	58	6.9
**Exchange absorbent materials at school**		
Yes	308	36.7
No	532	63.3
**Frequency of washing genitalia during menses**		
Once per day	217	25.8
More than twice per day	623	74.2
**Materials used for cleaning genitalia during menses**		
Soap and water	518	61.7
Water only	280	33.3
Plain paper	32	3.8
Others	10	1.2
**Materials used for washing hands**		
Water only	652	77.6
Soap and water	175	20.8
Others^a^	13	1.5
**Taking shower during menstruation**		
Yes	544	64.8
No	296	35.2
**A place where used absorbents disposed**		
Open field	87	10.4
Latrine	388	46.2
Waste bins/bury or burn it	327	38.9
Others	38	4.5

^a^Rags, paper and soft.

### School WASH facilities for MHM practice

Five hundred thirty-four (63.6%) adolescent girls had no access to clean water at school. Five hundred and thirteen (61.1%) girls had no access to toilet facilities at school. Four hundred and eighty-six (57.9%) students reported that the toilets do not contain a container to dispose the sanitary napkins. About half of the participants reported that their school did not have hand washing facilities (Table 3).

**Table 3:** Sociocultural and environmental factors for the management of menstrual hygiene in schools.

**Variable**	**Frequency**	**(%)**
**Had access to clean water**		
Yes	306	36.4
No	534	63.6
**Had access to toilet facilities at school**		
Yes	327	38.9
No	513	61.1
**Privacy of the toilet is kept**		
Yes	466	55.5
No	374	44.5
**Girls' toilet facilities separate from boys/ facilities**		
Yes	692	82.4
No	148	17.6
**Girls' individual toilet compartments lockable from the inside**		
Yes	474	56.4
No	366	43.6
**Girls' individual toilet compartments contain a container for disposing of napkins**		
Yes	354	42.1
No	486	57.9
**The school have hand-washing facilities**		
Yes	424	50.5
No	416	49.5
**Feel comfortable in school while menstruating**		
Yes	248	29.5
No	592	70.5
**Reason for being uncomfortable in school**		
No place to dispose used pad	153	22.4
No private place to change sanitary pad	242	35.43
No water for washing	162	23.72
Feeling of had Pain or discomfort	126	18.45
**Menstruation interferes school performance**		
Yes	266	31.7
No	574	68.3
**Activities restricted during menstruation**		
Yes	348	41.4
No	492	58.6

### Factors associated with menstrual hygiene management practice

The result of multiple logistic regressions analysis indicated that adolescents living with their parents had 2.51 higher odds of practicing safe menstrual hygiene compared to those living alone [AOR = 2.51, 95% CI: (1.11–5.68)]. Girls who lived with their relatives had 7.41 higher odds of practicing safe menstrual hygiene compared to those who lived alone [AOR = 7.41, 95% CI: (2.55–21.54)]. Girls whose mothers were merchants had 1.81 times higher odds of practicing safe menstrual hygiene compared to girls whose mothers were housewives [AOR = 1.81, 95% CI: (1.14–2.9)]. Girls whose mother's occupation status was private work were 4.56 times more likely to practice good menstrual hygiene practices than adolescents whose mothers were housewives [AOR = 4.56, 95% CI: (1.31–5.90)]. Girls whose fathers were farmers had 1.53 higher odds of practicing unsafe menstrual hygiene management compared to adolescent school girls whose fathers were merchants [AOR = 1.53, 95% CI: (1.1–2.31)]. Similarly, girls from rural areas had 1.61 higher odds of practicing unsafe menstrual hygiene management compared to those from urban areas [AOR = 1.61, 95% CI: (1.17–2.21)]. Girls who realized the lack of a container to store sanitary napkins in the toilet of the school latrine had 1.44 higher odds of practicing unsafe management of menstrual hygiene compared to their counterparts [AOR = 1.44, 95% CI: (1.1–1.94)] (Table 4).

**Table 4:** Factors associated with MHM for school girls of the north Shewa zone, high schools (multivariate analysis).

**Variables**	**Category**	**Unsafe MHM** ***n*** = **(%)**	**Safe MHM:** ***n*** = **(%)**	**COR** **(95%CI)**	**AOR** **(95%CI)**
Grade of students	Grade 9	62 (10.3%)	20 (8.4%)	1.1. (0.64–1.84)	1.24 (0.68–2.23)
	Grade 10	82 (13.6%)	37 (15.6%)	0.76 (0.49–1.2)	0.81 (0.5–1.32)
	Grade 11	144 (23.9%)	72 (30.4%)	0.69 (0.48–0.98)	0.68 (0.46–1.01)
	Grade 12	315 (52.2%)	108 (45.6%)	1	1
With whom do you live?	Both parents	407 (67.5%)	168 (70.9%)	2.24 (1.10–4.88)	2.51 (1.11–5.68)**
	Mother only	106 (17.6%)	42 (17.7%)		2.36 (0.99–5.64)
	Relatives	65 (10.8%)	9 (3.8%)	2.32 (1.01–5.35)	7.41 (2.55–21.54)**
	Father only	11 (1.8%)	5 (2.1%)	6.7 (2.4–18.74)	1.83 (0.45–7.40)
	Living alone	14 (2.3%)	13 (5.5%)	1	1
Occupational status of mother	Housewife	239 (39.6%)	127 (53.6%)	1	1
	Student	5 (0.8%)	3 (1.3%)	0.89 (0.21–3.77)	1.23 (.26–5.80)
	Merchant	115 (19.1%)	37 (15.6%)	1.65 (1.1–2.54)	1.81 (1.14–2.9)**
	Private work	25 (4.1%)	3 (1.3%)	4.43 (1.31–14.9)	4.56 (1.31–5.90)**
	Employee	53 (8.8%)	17 (2.2%)	1.66 (0.92–2.98)	1.59 (0.84–3.02)
	Daily laborer	70 (11.6%)	20 (8.4%)	1.86 (1.1–3.2)	1.56 (0.86–2.83)
	Farmer	96 (15.9%)	30 (12.7%)	1.7 (1.1–2.7)	1.40 (0.68–2.46)
Occupational status of father	Merchant	125 (20.7%)	63 (26.6%)	1	1
	Private work	26 (4.3%)	10 (4.2%)	1.31 (0.6–2.89)	1.65 (0.71–3.79)
	Employee	108 (17.9%)	40 (16.9%)	1.36 (0.85–2.18)	1.54 (0.91-2.61)
	Driver	27 (4.5%)	9 (3.8%)	1.51 (0.67–3.41)	1.94 (0.83–4.55)
	Daily laborer	120 (19.9%)	50 (21.1%)	1.21 (0.77–1.89)	1.46 (0.86–2.46)
	Farmer	197 (32.7%)	65 (27.4%)	1.53 (1.1–2.31)	1.84 (1.11–3.10)**
Residence	Urban	164 (27.2%)	89 (37.6%)	1	1
	Rural	439 (72.8%)	148 (62.4%)	1.61 (1.17–2.21)	1.62 (1.15–2.28)**
Individual toilet compartments contain a container for disposing of Napkins	Yes	239 (29.6%)	115 (48.5%)	1	1
	No	364 (60.4%)	122 (51.5%)	1.44 (1.1–1.94)	1.40 (1.1–1.92)**

COR, Curve Odds Ratio; AOR, Adjusted Odds Ratio; CI, Confidence Interval.

^**^The variable is a candidate for multivariate analysis at the value of p-value < 0.05, 1 = Reference group. The p-value of the fitness of the model (Hosmer and Lemshow) was 0.890.

### Findings from qualitative study

Twelve adolescent school girls participated in in-depth interviews. Six school directors and six girls club focal person teachers participated in key informant interviews. We stopped the in-depth interviews on 12 and the key informant interviews on 12 due to idea saturation. Four themes to explore barriers to menstrual hygiene management, and three themes emerged as enablers to menstrual hygiene management from the qualitative study. The themes include: lack of support from school, lack of awareness and knowledge about menstrual hygiene, lack of support from family, cultural aspects menstruation, living with parents, presence of functional girl club at school and presence of menstrual hygiene room in the school.

The finding from this study were discussed under three themes, living with parents, presence of menstrual hygiene room in the school, presence of functional girl club at school were identified as enablers to practice menstrual hygiene management.

**Theme one: Living with parents**

It was indicated that adolescent girls who live with their parents were happier to manage their menses as was said by one of in-depth interview participant who said:

“*I live with my parents and they knew all the information about MHM, the materials to absorb menstrual blood, and I want to thank them forever.”* (18-year female student)

*The other IDI participants said that:*

“*Currently, I live with my family. My mother and father are well educated, and they tell me everything related to menstrual hygiene practice... For instance, my mother told me how to use menstrual pads during menstruation.”*

**Theme two: Presence of functional girl club at school**

This study finding indicated that presence of functional girl club at school was identified that as enablers to practice menstrual hygiene management. According to one of the 19 years in-depth interview participants, the presence of functional female clubs in schools enhanced.

MHM.

“*I learnt more about MHM from our school girl clubs, and our girl club coordinator is my role model in sharing her experience and counseling us on MHM.”*

**Theme three: Presence of menstrual hygiene room in the school**

Different study participants said that presence of menstrual hygiene room in the school was encouraging the students to practice the menstrual management. It was indicated that students who learn at school which has house in which girls exchange absorbent materials were pleased to manage their menses as supported by a 17 years old girl:

“*I don't care or worry about menstruation at school because our school provided us with a single class where we could interchange menstrual absorbent materials and relax if we felt ill.”*

#### Barriers to practice menstrual hygiene managements

**Theme one: Lack of support from school**

Under this theme, four categories emerged, namely lack of a place where girls exchange absorbent materials, poor cleanliness of school latrines, lack of functional water source and lack of container to dispose used absorbent materials.

##### Lack of a place where girls exchange absorbent materials

Participants reported that lack of places where they exchange menstrual blood absorbent materials and lack of water affect their MHM at school. This was supported by an in-depth interview as one IDI participant stated:

'*When I menstruate, I never follow the teacher or participate in class activities in the classroom; rather, I am concerned about my period because there is no area where I can exchange modes and there is no water to wash at school.” (*19-year-old female student)

Another in-depth interview participant also claimed:

“*I am really concerned about the lack of a place where I exchange modes and lack of water to wash, for which I prefer to stay at home during menstruation even if my interest is not to miss school at any time.”* (17-year-old student)

##### Poor cleanliness of school latrines

As was reported by many of the participants, the students lack privacy in utilizing latrines and containers to dispose used absorbents as was stated below:

“*Our school's toilets lack privacy and containers to dispose of used absorbents; therefore, I choose not to change sanitary napkins at school or skip school 2–3 days per month.”* (16-year-old female student)

This was also supported by another in-depth interview participant who said:

”* we usually deposit the used absorbent materials in to the toilet because we didn't have any container; after two months, the toilet was filled because all females deposited used sanitary napkins in it, and now I hate going to school during my menstrual cycle because I don't have a place to change and dispose of absorbent materials at school.”* (15-year-old student)

This was also supported by another IDI participant who stated:

“*Because the school property and the latrine area are unclean and there is no water, the toilet is always dirty and unpleasant, making it unsafe to use or change modes.”* (14-year-old student)

##### Lack of functional water source and container to dispose used absorbent materials

From school observation we found that: The toilets lack a functioning water source, a container to dispose of absorbent materials, as well as a door and locks. The toilets were not clean enough, lack illumination and many of them were overflowing, full of feces, and difficult to use.

Another IDI participants said that:

“*In our school, we didn't have functional tap water to wash our hands and take showers... Most of the students disposed of the used pads in the field due to a lack of pad disposal containers.”* (15 years old student).

**Theme two: Lack of awareness and knowledge about menstrual hygiene**

Under this theme, one category emerged, namely lack of knowledge about menstrual hygiene.

##### Lack of knowledge about menstrual hygiene

It was reported that the lack of awareness about menses before reaching menarche affects preparation of girls to MHM as it was supported by the findings form an in-depth interview *17-year-old student* where one participant stated:

“*I will never forget what I experienced during my first menses; while sitting and learning in class, I noticed that blood flowed from my organ. I was terrified, confused, and fell from the bench to the ground, where my friends carried me out of the class while other students teased me and older girls told me that it was normal even if I did not believe them.”*

**Theme three: Lack of support from family**

Under this theme, one category emerged, namely lack of regular money to buy menstrual absorbents.

##### Lack of regular money to buy menstrual absorbents

Some of participants of this study reported that the possibility of getting regular money to buy absorbents affect their MHM. This is supported by a key informant interview who stated:

“*The majority of adolescents do not know the availability of commercial sanitary pads, since they do not receive money from anyone and most of them use rags to absorb menstrual blood.”* (36-year-old female teacher)

**Theme four: Cultural aspects menstruation**

Under this theme, one category emerged, namely menstrual related taboo and negligence.

##### Menstrual related taboo and negligence

Many of the study participants stated that the fact that menstruation is perceived as taboo affected their management of menstrual hygiene as was supported by findings from an in-depth interview, where some participants stated:

“*I was born and raised in rural areas where talking and discussing menstruation is taboo, which exposes me and my friends to unsanitary management of menstrual hygiene”*.

This is also supported by another in-depth interview participant who claimed:

“*I was neglected, limited to sitting on the couch, sleeping on the bed, and was seen as contaminated or dirty in my family while I was on menstruation.”* (17-year-old student)

## Discussions

The aim of this study was to assess safe menstrual hygiene management practice and associated factors among female adolescent students in public high schools in central Ethiopia.

Accordingly, the study revealed that the magnitude of safe menstrual hygiene management practice among adolescent schoolgirls was 28.2%. This finding is lower than the studies conducted in North East Ethiopia which indicated (51%) (25), Mehalmeda in Amhara, Ethiopia, (90.9%) (26), Lucy Village of Ethiopian Great Rift Valley, (70.2%) (19) Adama town, Ethiopia (57%) (21), Addis Ababa city, Ethiopia (51.3%) (18), Nekemte town, Ethiopia (39.9%) (33), Ambo town, Ethiopia (46.4%) (22), Dang District, Nepal (67%) (34), Kenya (71.2%) (14), Ghana (50.8%) (23), where the mentioned percentage of adolescent girls had good menstrual hygiene practice. The current finding is higher than the findings from Baherdir, Ethiopia which reported only 24.5% of adolescent girls practiced good MHM (27) and the study conducted in Uganda, where 90.5% of girls had poor MHM practice (32). The reason for the difference could be due to differences in sociodemographic characteristics, differences in study population as the majority of previous studies were conducted in urban areas whereas the current study was conducted in both urban and rural areas. Another possible explanation for the difference is that the way menstrual hygiene management was measured was different for the current study and previous studies.

In the current study, living with parents is an independent determinant of safe menstrual hygiene practice. This is consistent with findings from studies conducted in Dang District, Nepal (34) and Nigeria (35) where living with parents was positively associated with good menstrual hygiene practice. This could be because girls who live with their parents talk openly about their feelings and are better monitored and directed throughout their menstrual cycle than those who live alone. This finding was supported by qualitative study results. It was indicated that adolescent girls who live with their parents were happier to manage their menses as stated IDI. The finding of this study also found a positive association of MHM and the lives of adolescent girls with their families. The possible explanation for this fact is that girls who live with their relatives are more likely to be aware of menstrual hygiene and to obtain money to buy menstrual blood absorbents than those who live alone.

Adolescent girls whose mothers are merchants have a higher chance of practicing safe menstrual hygiene than those whose mothers are housewives. This is related to the issue of family income so that mothers who are merchants may have improved income to buy the necessary materials necessary to practice safe MHM for their daughter. It is supported by the finding from qualitative studies. Some of participants of this study reported that the possibility of getting regular money to buy absorbents affects their MHM as stated by KII participating teachers.

Adolescent girls whose mothers have private work have a higher chance of practicing safe menstrual hygiene than those whose mothers are housewives. It is supported by the finding from qualitative studies. Some of participants of this study reported that the possibility of getting regular money to buy absorbents affects their MHM as stated by KII participating teachers. The possible explanation might be that mothers who have private work may have their own income to give permanent pocket money to their adolescent girls to buy materials such as menstrual blood absorbents.

Adolescent girls, whose fathers were farmers, are more likely to practice unsafe menstrual hygiene. It is consistent with a study conducted in Ghana where the farmer's occupation father was positively associated with poor MHM (23). This is due to the fact that girls in agricultural households use inferior or unclean menstrual absorbents and do not have access to hygienic facilities for menstruation management (36). Furthermore, girls from the farmer family are from the country side, where there is not enough information on MHM, leading to unsafe MHM.

This study revealed a positive association between living in rural areas and unsafe menstrual hygiene practices. It is similar to studies from North East Ethiopia (25) and Mehalmeda in Amhara, Ethiopia (26) where Urban place of residence was positively associated with good menstrual hygiene management practices. This is because rural areas do not have access to information on menstrual hygiene compared to urban residents. This finding is supported by the findings of a qualitative study. The majority of the rural community perceives menstrual hygiene management as a taboo and a neglected topic, which leads to poor practice.

The absence of a container for the storage of sanitary napkins in the school latrine toilet had a positive association with unsafe management of menstrual hygiene management. It is similar to the study conducted in Zambia which found a positive association between the lack of WASH facilities at school and poor MHM (37). This is because as girls lack a place where to dispose of absorbent materials used in schools, they are exposed to unsafe menstrual hygiene and feel uncomfortable. The qualitative result supported this finding. According to the findings of school observations and IDI participants, the main reasons for poor menstrual hygiene management practice was a lack of a functioning water source, a container to dispose of absorbent materials, as well as a door and locks.

### Strength of the study

Using mixed-methods approach, consisting of quantitative and qualitative studies together.

### Limitation of the study

Because menstruation hygiene is considered a sensitive topic to discuss, the study could be prone to bias toward social desirability. Because it is cross-sectional research, it is difficult to determine the true relationship between menstrual hygiene management and independent factors.

## Conclusions

The safe management of menstrual hygiene management practices among adolescent school girls was low. People with whom adolescent girls live, the occupational status of mother, the occupational status of father, residence, and the availability of containers to dispose of sanitary napkins in school toilets were factors associated with the management of menstrual hygiene. To overcome this problem, effort is needed from health promoters, school leaders, government and non-government organizations. The government should give attention to school-based menstrual hygiene management room establishments. Health professional should communicate behavioral changes in menstrual hygiene management to female students and the community. The school should have containers to dispose of the menstrual blood absorbent materials used in the girls' toilets at school.

## Data availability statement

The raw data supporting the conclusions of this article will be made available by the authors, without undue reservation.

## Ethics statement

The studies involving human participants were reviewed and approved by Salale University Ethical Review Committee. Written informed consent to participate in this study was provided by the participants' legal guardian/next of kin.

## Author contributions

All authors listed have made a substantial, direct, and intellectual contribution to the work and approved it for publication.

## Funding

This research work was funded by Salale University. The funders had no role in study design, data collection, analysis, decision to publish, or manuscript preparation.

## Conflict of interest

The authors declare that the research was conducted in the absence of any commercial or financial relationships that could be construed as a potential conflict of interest.

## Publisher's note

All claims expressed in this article are solely those of the authors and do not necessarily represent those of their affiliated organizations, or those of the publisher, the editors and the reviewers. Any product that may be evaluated in this article, or claim that may be made by its manufacturer, is not guaranteed or endorsed by the publisher.

## Abbreviations

IDI: In-depth interview
KII: Key informant interview
MHM: Menstrual Hygiene Management
SDG: Sustainable Development Goal
UNICEF: United Nations Children's Fund
WASH, Water: Sanitation and Hygiene
WHO: World Health Organization.
